# Neurorehabilitation with vagus nerve stimulation: a systematic review

**DOI:** 10.3389/fneur.2024.1390217

**Published:** 2024-05-30

**Authors:** Radha Korupolu, Alyssa Miller, Andrew Park, Nuray Yozbatiran

**Affiliations:** ^1^University of Texas Health Science Center at Houston, Houston, TX, United States; ^2^TIRR Memorial Hermann Hospital, Houston, TX, United States; ^3^Craig Hospital, Englewood, CO, United States; ^4^University of Colorado Hospital, Aurora, CO, United States

**Keywords:** vagus nerve stimulation, stroke, brain injury, spinal cord injury, rehabilitation, motor activity vagus nerve stimulation, spinal cord injury stroke, motor activity

## Abstract

**Objective:**

To systematically review vagus nerve stimulation (VNS) studies to present data on the safety and efficacy on motor recovery following stroke, traumatic brain injury (TBI), and spinal cord injury (SCI).

**Methods:**

Data sources: PubMed, EMBASE, SCOPUS, and Cochrane.

**Study selection:**

Clinical trials of VNS in animal models and humans with TBI and SCI were included to evaluate the effects of pairing VNS with rehabilitation therapy on motor recovery.

**Data extraction:**

Two reviewers independently assessed articles according to the evaluation criteria and extracted relevant data electronically.

**Data synthesis:**

Twenty-nine studies were included; 11 were animal models of stroke, TBI, and SCI, and eight involved humans with stroke. While there was heterogeneity in methods of delivering VNS with respect to rehabilitation therapy in animal studies and human non-invasive studies, a similar methodology was used in all human-invasive VNS studies. In animal studies, pairing VNS with rehabilitation therapy consistently improved motor outcomes compared to controls. Except for one study, all human invasive and non-invasive studies with controls demonstrated a trend toward improvement in motor outcomes compared to sham controls post-intervention. However, compared to non-invasive, invasive VNS, studies reported severe adverse events such as vocal cord palsy, dysphagia, surgical site infection, and hoarseness of voice, which were found to be related to surgery.

**Conclusion:**

Our review suggests that VNS (non-invasive or invasive) paired with rehabilitation can improve motor outcomes after stroke in humans. Hence, VNS human studies are needed in people with TBI and SCI. There are risks related to device implantation to deliver invasive VNS compared to non-invasive VNS. Future human comparison studies are required to study and quantify the efficacy vs. risks of paired VNS delivered via different methods with rehabilitation, which would allow patients to make an informed decision.

**Systematic review registration:**

https://www.crd.york.ac.uk/prospero/display_record.php?RecordID=330653.

## Introduction

Approximately one million people suffer from stroke, traumatic brain injury (TBI), and spinal cord injury (SCI) in the United States alone, resulting in significant disability due to loss of functional abilities required to live independently, such as transfers and activities of daily living (1–5). Specifically, severe impairment in arm/hand motor function requires significant assistance and caregiver support, resulting in enormous lifetime direct and indirect costs (6–12). Various approaches to improve upper extremity motor function after stroke, TBI, and SCI include standard rehabilitation therapy focusing on strengthening, stretching, and task-specific movement therapy with or without biofeedback, robotic therapy, functional electrical stimulation, constraint-induced movement therapy, and reconstructive surgeries (13–20). However, recovery is challenging; even after completing conventional rehabilitation therapies, people frequently have residual motor disabilities following stroke, TBI, and SCI. It is clear that additional facilitation of neuroplastic change is required to achieve a drastic shift in the rehabilitation status quo (21).

Neuroplasticity is the capacity of spared neural cells and pathways to change in response to intrinsic and extrinsic factors aiding motor recovery after neurological injury (22–24). Hence, in the last decade, there has been an interest in pairing rehabilitation therapy with various neuromodulation interventions, including vagus nerve stimulation. The vagus nerve can be stimulated via external electrodes placed over either the auricular branch or cervical branch non-invasively or invasively via direct electrode placement over a cervical branch of the vagus nerve over the anterior aspect of the neck. The pairing of vagus nerve stimulation (VNS) with movement or sensory input for generating targeted neuroplasticity has demonstrated a potential for clinical application (25–34). The vagus nerve is an important cranial nerve that carries parasympathetic and brachial motor efferents to several target organs, and a large proportion of vagus nerve fibers also consists of afferent connections to several nuclei in the brain stem. These connections regulate the release of neuromodulators, including acetylcholine, norepinephrine, serotonin, and brain-derived neurotrophic factors, which promote cortical plasticity (30, 31, 35–39).

Several studies have been conducted in animals and humans to explore the promising effects of VNS on motor recovery following a neurological injury. However, there is heterogeneity in the study population, mode and location of VNS, stimulation parameters, timing of stimulation when combined with rehabilitation therapy, and lack of consistent reporting on safety and adverse events. It is essential to systematically analyze and review pre-clinical and clinical data to understand the underlying mechanism, principles, and safety; to identify the gaps to fill prior to rapid clinical translation of VNS for motor recovery. In one of the recently published systematic reviews on VNS in the stroke population (40), results from non-invasive and invasive studies were combined when the meta-analysis was performed. It is not ideal to combine study results from non-invasive and invasive VNS studies due to differences in methods of stimulation and mechanisms; the experts in the field of VNS expressed similar views on this review paper in a recent publication (41).

To fill these gaps in the literature, we performed an overarching complete review of the safety and efficacy of VNS on motor recovery following neurological injury in animals and humans.

We performed a quantitative synthesis of primary motor outcomes reported in invasive and non-invasive VNS human RCTs, separately utilizing a novel method recently published in two prestigious journals, which provides an estimate of relative improvement in the intervention group compared to controls. The relative improvement is a critical estimate, allowing readers to compare improvements of non-invasive and invasive VNS, and to determine if the additional benefit is worth the risk associated with surgical implantation of VNS devices based on current evidence. Finally, we provided a detailed account of adverse events reported in these studies and the cumulative incidence of each type of adverse event when data was available.

## Methods

This systematic review followed the preferred reporting items for systematic reviews and meta-analyses (PRISMA) guidelines. It was pre-registered with the PROSPERO prospective register of systematic reviews (CRD42022330653).

### Search strategy and inclusion criteria

We conducted a literature search on PubMed, EMBASE, SCOPUS, and Cochrane Central Register of Controlled Trials using search terms: “vagus nerve stimulation,” “stroke,” “brain injury,” and “spinal cord injury.” We included all clinical trials (both randomized and non-randomized) of vagus nerve stimulation in animal models and humans with stroke, TBI, and SCI published between 1 January 2002 and 15 May 2022, focusing on the effects of VNS on motor recovery.

### Data extraction

**Figure 1:**
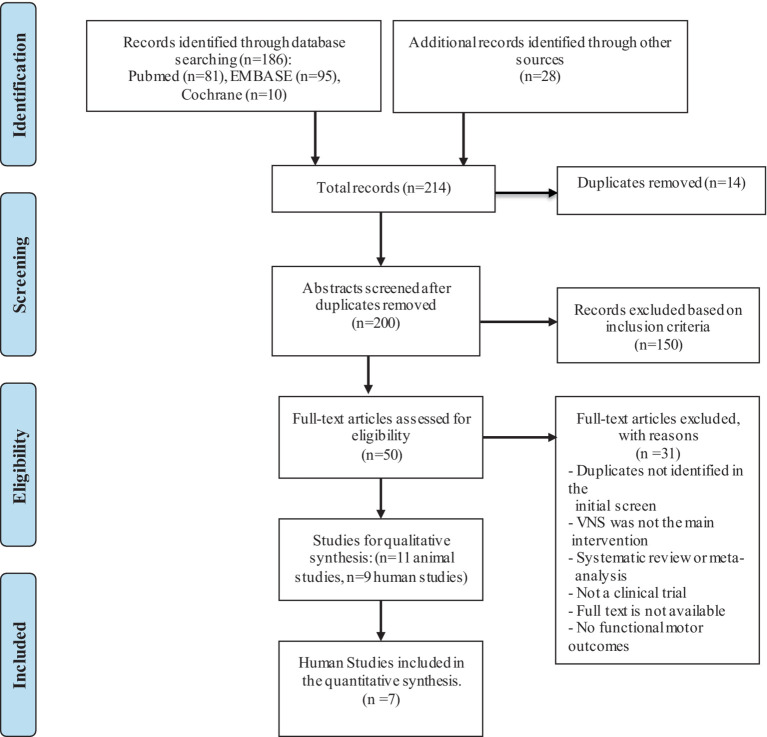
PRISM flow diagram.

Two authors independently reviewed all abstracts found from the above search strategy and screened the abstracts for eligibility using similar criteria for animal and human studies. After removing duplicates, two authors (RK and AM) reviewed 200 abstracts and excluded studies that did not meet eligibility criteria (Figure 1). After identifying eligible papers through abstract review, authors RK, AM, and NY reviewed full texts for final inclusion and data extraction. We also excluded studies that did not assess the effects of VNS on motor recovery and did not report any motor or functional outcomes as primary or secondary outcome measures. Desired data was extracted in electronic data collection forms. Data elements included detailed information on demographics, diagnosis, study design, intervention, controls, and outcome measures (Tables 1, 2). We also collected detailed information on the effects of VNS compared to controls on motor and functional outcome measures.

**Table 1:** Invasive VNS animal studies.

Study and study design	Animal species/Gender	Lesion	Sample size	Control	Active intervention	Frequency and duration	Motor outcomes measures (T1 = baseline, T2: post-intervention, T3: f/u)	Results-motor outcomes
**Stroke studies**								
Khodaparast, 2013 (control trial)	4 months old Female Sprague–Dawley Rats	Unilateral motor cortex ischemic lesion of M1	Paired VNS = 9 Control = 9	Only RT: Isometric force task training	Paired VNS to left CVN, delivered on successful trials with RT	30 min twice daily or 5 days/week for 5 weeks	Peak force, Hit rate at T1, T2 and T3 at 1 week	Paired VNS vs. control +S at T2 and T3
Khodaparast, 2014 (control trial)	Adult Female	Unilateral ischemic lesion -left M1	Paired VNS = 8 Control = 9 Delayed VNS = 3	Only RT: Bradykinesia task	Paired VNS to left CVN stims delivered on successful trials with RT	30 min twice daily for 5 days/week for 5 weeks	Hit rate at T1 and T2	Paired VNS vs. control: +S Delayed VNS vs. control: 0
	Sprague–Dawley Rats				Delayed VNS (1): every 12 s for 1 h received 2 h after RT			
Hays, 2014 (control trial)	4 months old Female Sprague–Dawley Rats	Unilateral motor cortex ischemic lesion of M1	Paired VNS = 8 Extra VNS = 6 Delayed VNS = 7 Control: 10	Only RT: Isometric force task training	Paired VNS to left CVN on successful trials with RT	30 min twice daily for 5 days/week for 5 weeks	Maximum pull force and hit rate at T1 and T2.	Paired VNS vs. control: +S Extra VNS vs. control: +S Delayed VNS vs. control: +S Paired VNS superior to delayed and Extra VNS (+S)
					Delayed VNS: every 10 s for 1 h, 2 h after RT			
					Extra VNS: VNS during RT at an average every 2 s			
Hays, 2014 (control trial)	Female Sprague–Dawley rats	Left intracerebral hemorrhage	Paired VNS = 14 Control = 12	Only RT: Bradykinesia task	Paired VNS on successful trials	30 min twice daily for 5 days/week for 6 weeks	Hit Rate at T1 and T2	VNS (paired + extra) vs. control: +S Paired VNS vs. Extra VNS:0
					Extra VNS: received VNS on all trials			
Khodaparast, 2015 (control trial)	Four months old Female	Unilateral ischemic lesions of the primary motor cortex	Paired VNS = 10	Only RT: Isometric force task training	Paired VNS to left CVN	30 min twice daily for 5 days/week for 6 weeks	Maximal pull force, hit rate at T1 and T2	Paired VNS vs. control: +S Paired VNS vs. delayed VNS: +S Delayed VNS vs. control: 0
	Sprague–Dawley Rats		Delayed VNS = 10					
			Control = 9		Delayed VNS: VNS every 12 s for 1 h after RT			
Hay, 2016 (control trial)	18 months old aged female fisher rats	Unilateral motor cortex ischemic lesion of M1	Paired VNS = 8 Control = 9	Only RT: Isometric force task training	Paired VNS to left CVN delivered on successful trials	30 min twice daily for 5 days/week for 5 weeks	Peak pull force, hit rate at T1 and T2	Paired VNS vs. control: +S
Meyers, 2018 (RCT)	Adult female Sprague–Dawley rats	Unilateral motor cortex ischemic lesion of M1 and dorsolateral striatum	Paired VNS = 9 Control = 10	Only RT: supination task, isometric pull task	Paired VNS to left CVN delivered on successful trials	30 min twice daily for 5 days/week	Peak turn angle, peak pull force at T1, T2 and T3–6 weeks f/u	Paired VNS vs. control: +S at T2 and T3
						for 5 weeks		
Pruitt, 2021 (control trial)	Adult female Sprague–Dawley rats	Unilateral traumatic lesion of left motor cortex	Paired VNS = 14 Control = 14	Only RT: Isometric pull task training	Paired VNS to left CVN delivered on successful trials	30 min twice daily for 5 days/week for 5 weeks	Maximal pull force, hit rate at and T2	Paired VNS vs. control: +S
Jiang, 2016 (RCT)	Male Sprague–Dawley rats	Unilateral focal ischemic lesions	I/R + aVNS = 8 I/R = 8 I/R + Sham aVNS =8	No RT	aVNS over left cavum concha via acupuncture needles	30 min twice daily for 5 days/week for 3 weeks	Beam-walking test and staircase test at T1 and T2	aVNS vs. I/R: +S avNS vs. I/R+ sham: +S
**Spinal cord injury**								
Ganger 2018 (control trial)	Adult female Sprague Dawley rats	Right Unilateral or bilateral C6 traumatic lesion	Top 20% CLV =13 Bottom 20% CLV = 8 Control = 9	Only RT: Isometric pull task training	Paired VNS to left CVN on successful trials during RT	F: 30 min twice daily for 5 days/week for 5 weeks	Peak pull force at T1 and T2	Top 20% CLV vs. control: +S Bottom 20% CLV vs. control: 0
Darrow 2020 (control trial)	Adult female Sprague Dawley rats	Bilateral contusive SCI C7/C8/ 9 wks	Paired VNS = 10 Control = 8	Only RT: Isometric pull task training	Paired VNS to left CVN delivered when a pre-set threshold was met for pull force during RT	30 min twice daily for 5 days/week	Peak force, hit rate at T1 and T2	Paired VNS vs. control: +S
						for 5 weeks		

+S indicates a statistically significant improvement. (*p* < 0.05) in the active intervention group/VNS compared to the control group; 0 indicates no significant difference in outcomes between groups. aVNS, Auricular vagus nerve stimulation; CLV, closed loop vagus nerve stimulation; CVN, Cervical vagus nerve; f/u, follow-up; I/R, infusion and reperfusion; RT, Rehabilitation therapy; VN, Vagus nerve.

**Table 2:** Human stroke VNS studies demographics and baseline.

Study and study design	Population	Age in years, mean (SD)	Sample size, % male (M)	Time since stroke in months, mean (SD) or Median (IQR)	Interventions in respective groups (I, intervention; L, location of VNS; T, timing of VNS; F, frequency; CI, current Intensity)	Duration (D) and follow-up (f/u) (T1, baseline; T2, post-intervention; T3, follow-up)	Outcomes
**Non-invasive VNS studies**							
Capone et al. 2017/RCT	Chronic Ischemic or hemorrhagic stroke	I: 54 (5.9)	I: 7 (M: 57%)	I: 94 (39)	I: tVNS + UE robotic therapy to the affected limb	D: 10 days	Motor
					L & T: Left inner side of the tragus, before robotic therapy, every 5 min for 1 h		FMA-UE: +S
					F & CI: 30 Hz and CI range 2–4.5 mA	F: >300 point-to-point movements per session	Adverse events: No adverse events occurred, no change in BP and HR
		C: 56 (7.1)	C: 5 (M:43%)	C: 46.0 (22)	C: sham tVNS + Robotic therapy	OA: T1 and T2	
Redgrave et al. 2018/Pre-post study	Chronic ischemic stroke	65 (6.9)	13 (M:77%)	14 (IQR: 8–43)	I: tVNS + UE rehabilitation therapy to the affected limb	D: 1 h RT with sham or active tVNS, three times per week for 6 weeks	Motor
							FMA-UE: Improved pre-post^#^
					L & T: Left concha, paired with RT	F: >300 repetitions per hour session	ARAT: Improved pre-post^#^
					F & CI: 25 Hz and median (IQR) CI 1.4 (1–3.2) mA	OA: T1 and T2	Adverse events: Two reported fatigue, one lightheadedness, no change in ECG.
Wu et al. 2020/RCT	Sub-acute Ischemic stroke	I: 65 (9.9)	I: 5 (M:50%)	I: 1.2 (0.31)	I: taVNS pre-RT followed by RT	D: 30 min sham or taVNS +30 min RT daily for 15 days	Motor
							FMA-UE: +S at T2 and T3
							WMFT: +S at T2 and T3
					L & T: Left cymba concha, pre-RT	F: multiple movements were targeted until fatigue	FIM: +S at T2 and T3
					F & CI: 20 Hz and mean (SD) CI 1.7 (0.4) mA	OA: T1 and T2, T3–12 weeks from 1st intervention	
		C: 62 (11)	C: 8 (M:73%)	C: 1.2 (0.22)	C: Sham taVNS + RT		Adverse events: One episode of skin redness, no group differences for HR & BP.
Chang et al. 2021/RCT	Chronic ischemic or hemorrhagic stroke	I: 56	I: 18 (M:50%)	26 (4.7)	I: taVNS + UE robotic therapy	D: 1 h/session	Motor
					L & T: Left cymba concha paired with robotic therapy	F: 3 ×/week for 3 weeks	FMA-UE: 0 at T2 and T3
							WMFT: 0 at T2 and T3
							MRC: 0 at T2 and T3
							MTS: 0 at T2 and +S at T3
				Only cumulative data is available	F & CI: 30 hz, 0.1–5 mA	OA: T1, T2 and T3:3 months f/u	^*^Both groups improved pre-post but no statistical difference was seen between groups except for MTS at f/u.
		C: 62	C:15 (M:50%)		C: Sham taVNS + UE robotic therapy		Adverse events: No serious adverse events were reported in this study.
Li et al. 2022/RCT	Acute ischemic or hemorrhagic stroke	I: 69(12)	I: 30 (M: 50%)	I: 0.36 (0.25)	I: tavNS + RT	D: 20 min sham or active taVNS +30 min RT/session	Motor
					L & T: Left concha, pre-RT for 20 min	F: 5×/week for 4 weeks	FMA-UE, FMA-LE, FMA-S: +S at T2 and all f/u assessments
				C: 0.34(0.23)	F & CI: 20 Hz, mean (sd): 1.7(0.5) mA	OA: T1, T2, T3:3,6 and 12 months from baseline	WMFT: +S at T2 and all f/u assessments
		C: 68(12)	C:30 (M:47%)		C: Sham taVNS + RT		Adverse events: No changes in HR SBP and DBP pre-post therapy in each group. No adverse events.
**Invasive VNS studies**							
Dawson et al. 2016/RCT	Ischemic stroke	I:58 (17)	I: 9 (M: 78%)	I:14 (12)	I: active VNS + RT	D: 2 h per session	Motor:
							FMA-UE: +S (per protocol analysis)
							FMA-UE: 0 (intent to treat analysis)
					L & T: Left cervical vagus nerve paired stim for 2 h	F: 3 ×/week for 6 weeks	ARAT, grip strength, NHP, BBT: 0 (per protocol analysis)
							Adverse events in VNS (n):
							*Surgical complication:*
							Vocal cord palsy and dysphagia (1),
					F & CI: 30 Hz, 0.8 mA	OA: T1 and T2	Taste disturbance (1),
		C:61(11)	C:11 (M:82%)	C:20 (16)	C: RT only		*Stimulation-related events:*
							Hoarseness of voice or neck tingling (6),
							Nausea after a single session (1)
							Difficulty swallowing after one VNS session (1)
Kimberley et al. 2018/RCT	Ischemic stroke	I: 60 (7)	I:8 (M:50%)	I: 18	I: active VNS + RT + home exercise	In clinic therapy:	Motor:
							FMA-UE, BBT, NHPT: 0 at T2 and T3
							WMFT: 0 at PI; +S at T3
							Adverse events in VNS:
						D: 2 h per session in clinic therapy	*Surgical complication:*
					L & T: Left cervical vagus nerve paired stim for 2 h	F: 3 x/week for 6 weeks	Surgical site infection (1)
				^*^SD unknown	F & CI: 30 Hz, 0.8 mA	Home Exercise:	Post-surgical SOB and dysphagia (1)
		C: 60 (14)	C:9 (M:56%)	C: 18	C: Sham VNS + RT only + home exercise	30 min daily for 90 days	Hoarseness of voice due to vocal cord palsy (1)
				^*^SD unknown		OA: T1, T2, and T3: 90 days f/u	^*^No serious adverse events associated with VNS were reported.
Dawson et al. 2021/RCT	Ischemic stroke	I: 59 (10)	I: 53 (M:64%)	I: 37 (28)	I: active VNS + RT + home exercise	In-clinic therapy:	Motor:
							FMA-UE: +S at T1 and T2
							WMFT: +S at PI; +S at T1 and T2
							Serious adverse events in VNS:
						D: Avg 90 min/session in clinic therapy	*Surgical complications:*
						F: 3 ×/week for 6 weeks	Surgical site pain, *n* = 24(22%)
					L & T: Left cervical vagus nerve paired stim for 2 h	Home Exercise:	Hoarseness of voice, *n* = 9 (8%)
					F & CI: 30 Hz, 0.8 mA	30 min daily for 90 days	Vocal cord palsy, *n* = 1 (0.01%)
		C: 61 (9)	C:55 (M:65%)	C:40 (31)	C: Sham VNS + RT only + home exercise	OA: T1, T2 and T3: 90 days f/u	^*^No serious adverse events associated with the device stimulation were reported.

+S indicates a statistically significant improvement (*p* < 0.05) in the active intervention/VNS group compared to the control group; 0 indicates no significant difference between groups, ^#^Statistical analysis was not performed. FMA-LE, Fugl-Meyer Assessment—lower extremity; FMA-S, Fugl-Meyer Assessment –sensory; MAL, Motor activity log; MRC, Medical Research Council Motor Power Scale; MTS, Modified Tardieu Scale; NHPT,: Nine Hole Peg Test; SBP, Systolic blood pressure; DBP, Diastolic blood pressure; RT, Rehabilitation therapy; OA, Outcome assessment.

### Data synthesis

We provided a qualitative description of the effects of VNS on outcomes and summarized the data separately for animal and human studies in Tables 1, 2, respectively. We also included the results of primary outcome data from human RCTs in Table 3. We reported the results of all functional motor outcomes in Tables 1, 2 if provided in the literature. We summarized the results in tables based on a previously used method by our research team in a recent systematic review. The characters “+S” or “0” were used to indicate whether a particular outcome measure achieved statistical significance, with “+S” in favor of VNS therapy, or “0” if there was no difference between active VNS and control groups (42). We did not perform a meta-analysis due to high variability in the methods used for VNS, and our team considered it would not provide an accurate effect size (43). VNS experts expressed similar concerns about the inappropriateness of combining results from various stimulation methods, published in a commentary (41). Hence, we calculated the relative change in outcome measures to assess the efficacy of VNS therapy on the most widely used upper extremity motor outcome measure, the upper extremity Fugl-Meyer-Assessment (FMA-UE), separately for non-invasive and invasive studies utilizing methods in two recently published systematic review papers (42, 44).

**Table 3:** Results-mean improvement in upper extremity Fugl-Meyer scores in people with stroke.

	VNS group, mean (SD)	Control group, mean (SD)	Mean improvement in FMA-UE scores (CI)	*p*-value	Risk of bias
**Non-invasive VNS studies**					
Capone et al. 2017/RCT	5.4 (7.2)	2.8 (7.1)	2.6^*^	0.048	Some
Wu et al. 2020/RCT	6.9 (1.9)	3.2 (1.2)	3.7 (2.3 to 5.1)	≤0.001	Low
Chang et al. 2021/RCT	3 (0.57)	2.9 (0.5)	0.14^*^	≥0.23	Some
Li et al. 2022/RCT	13^*^	4.7^*^	8.3^*^	<0.05	Some
**Invasive VNS studies**					
Dawson et al. 2016/RCT	8.7 (5.8)	3 (6.1)	5.7 (−0.36 to 12)	0.064 (ITT)	Some
	9.6 (5.3)	3 (6.1)	6.5 (0.42 to 12.6)	0.038 (PP)	
Kimberley et al. 2018/RCT	7.6*	5.3^*^	2.3 (−1.9 to 6.5)	0.26 (ITT)	Low
Dawson et al. 2021/RCT	5 (4.4)	2.4 (3.8)	2.6 (1 to 4.2)	0.0014	Low

^*^SD was not provided in the original paper. CI, Confidence interval; ITT, Intention to treat analysis; PP, Per protocol analysis.

The relative change for outcome measure FMA-UE was obtained after subtracting the pre-post change in FMA-UE score in the active VNS group from the pre-post change in FMA-UE score in the control group, divided by the average baseline FMA-UE score of the VNS group. This relative difference or change estimates the percentage improvement or worsening of the FMA-UE score in the VNS group relative to the control group. We also calculated the median (IQR) for the mean improvement in upper extremity-FMA scores in the VNS group compared to the control group for human studies.

### Risk of bias

Human studies included in relative change calculations were appraised for risk of bias using the ROBIN-II tool for RCTs (45). Detailed appraisal report is outlined in Supplementary material and the overall risk of bias for the appraised studies is reported in Table 3.

## Results

Based on our literature search criteria, we identified 11 VNS studies on motor recovery in animal models and 8 in humans (Figure 1). Among animal studies, eight (73%) were in stroke models, one in TBI (9%), and 2 (18%) were in SCI models (Table 1). All eight human studies were conducted in people with stroke. During our literature search, there were no studies published involving VNS on motor recovery in people with SCI and TBI. We categorized our review and summarized results in the following categories: (1) VNS studies in animals with stroke models, (2) VNS studies in animals with SCI models, and (3) VNS studies in humans with stroke, further categorized in non-invasive and invasive studies.

### VNS studies in animals with the stroke model

Nine studies met the eligibility criteria for our review (Table 1) (29, 31, 32, 46–51). Among these, seven studies were conducted in animal models of ischemic lesions to the motor cortex, one study (46) in animal models with unilateral intra-cerebral hemorrhage, and one study in animal models of the unilateral traumatic lesion to the left motor cortex (51). Eight of the studies (89%) implanted electrodes to stimulate the left cervical vagus nerve, and in one study (47) the left auricular vagus nerve was stimulated using acupuncture needles.

#### Invasive cervical vagus nerve studies

Female Sprague–Dawley Rats were used to study the effects of VNS in these studies. Prior to induced stroke or brain injury, all animals were trained to the respective rehabilitation tasks per protocol. In most studies, the intervention duration was 5 weeks; in two studies, the intervention duration was 6 weeks (Table 1). While most studies compared the effects of pairing VNS with rehabilitation compared to rehabilitation therapy, a few studies also explored the effects of delayed VNS and extra VNS (Table 1). In the paired VNS studies, the VNS group received VNS during rehabilitation therapy immediately after a successful trial at a 30 Hz frequency. In the three studies with delayed VNS group, VNS was delivered after 2 h of rehabilitation therapy every 10–12 s for 1 h (32, 46, 48). The delayed VNS protocol often optimized stimulation frequency to match the number of stimulations received by animals in paired VNS groups. The extra VNS group in two studies (29, 46) received additional VNS compared to paired VNS, delivered at a higher frequency for the same duration. The control group received identical rehabilitation therapy for the same duration as the paired VNS group without any active VNS (Table 1). Most studies performed follow-up assessments a week after the completion of the intervention. In all eight studies, paired VNS was delivered at a current intensity of 0.8 mA with 100 μs phase duration.

Across all eight studies, measured motor outcomes improved significantly in the paired VNS group compared to the control group. Extra VNS resulted in improvement in motor outcomes compared to control which was statistically significant but did not result in any additional benefit compared to paired VNS. However, in studies with delayed VNS groups, results were inconsistent. In two studies (32, 48), there were no differences in outcomes between delayed VNS and controls; in one study (29), the delayed VNS group had better motor outcomes than the control, but the paired VNS group had statistically significantly improved motor outcomes compared to the extra VNS and delayed VNS group. There was no difference in lesion size pre and post-intervention based on histological processing in all eight studies. None of the above studies reported or recorded any adverse events during the study procedures.

#### Auricular vagus nerve study in stroke

In this study, male Sprague–Dawley rats with unilateral focal ischemic lesions of the motor cortex, were randomized into three groups (47). One group received only reperfusion therapy, a second group received both reperfusion therapy and stimulation of the auricular branch of the vagus nerve over the left cavum concha with acupuncture needles, and a third group received reperfusion therapy, with acupuncture needles implanted, but VNS stimulation was not delivered. In the second group, VNS was delivered at 0.5 mA current intensity at 20 Hz every 5 mins for 1 h. None of the groups received rehabilitation therapy. Functional recovery was measured by the beam-walking test and staircase test. At 1 week follow up, these tests revealed a statistically significant improvement in neurological function in the active VNS group with reperfusion compared to the other two groups, which persisted at the third week. Additionally, the size or volume of the infarct significantly reduced in the active VNS group compared to the other two groups.

### VNS studies in animals with SCI model

Only two studies in animal models of SCI met the eligibility criteria for our review (25, 52). Both studies were performed in female Sprague–Dawley rats, and in both studies animals sustained traumatic lesions at the cervical level (Table 1). Electrodes were directly implanted on the left cervical vagus nerve for stimulation. Similar to stroke studies, animals were trained before traumatic cervical SCI. The intervention duration was 5 weeks, and stimulation duration and parameters were identical to stroke animal studies at a current intensity of 0.8 mA, frequency of 30 Hz, and 100 μs phase duration (Table 1). In both studies, rats received isometric pull task training paired with or without VNS (control). Outcomes were measured 1 week after completion of the intervention.

In one study (52), animals were divided into three groups: (a) a control group received rehabilitation without VNS, (b) the top 20% closed loop VNS (CLV) group in which VNS was delivered immediately after an isometric pull task trial when pull force was within the top 20% of prior trials and (c) the bottom 20% CLV group in which VNS was delivered in which pull forces fell within the bottom 20% of the prior trials resulting in a significant time gap between VNS and successful trials. In this study, the top 20% of the CLV group had improvement in peak pull force post-intervention compared to the control group, which was statistically significant, but the bottom 20% of the CLV group and control group had no difference in peak pull force compared to controls. In the second study, pairing VNS with rehabilitation resulted in a statistically significant improvement in forelimb strength measured by peak pull force and hit rate compared to the control group (25). Adverse events were not documented in either study.

### VNS studies in humans with stroke

Among eight human studies, five (63%) utilized a non-invasive mode of VNS, and three (37%) studies involved surgical implantation of electrodes on the cervical portion of the vagus nerve to deliver VNS.

Among the seven RCTs included in the quantitative evaluation (Table 3) of outcomes, 3 (43%) were found to have low risk, 4 (57%) some risk of bias (Supplementary material 1), and 0 (0%) high risk of bias.

#### Non-invasive VNS human stroke studies

Among five non-invasive studies, three studies (53–55) included patients with chronic stroke (occurring 6 months prior), one study (56) was done in sub-acute stroke (>1 month and <3 months since stroke), and one study (57) in acute stroke patients (stroke onset < 1 month). Four of the five studies were randomized, blinded RCTs receiving active transcutaneous auricular VNS (taVNS) or sham VNS. The fifth study was a single group examining the effects of taVNS pre and post-intervention. In all studies, the left auricular branch of the vagus nerve was stimulated transcutaneously with surface electrodes. However, specific placement varied slightly between studies (Table 2). In two studies, taVNS was paired with rehabilitation therapy, each lasting up to 1 h. In the other three studies, taVNS was delivered before rehabilitation therapy, ranging from 20 min to 60 min. Stimulation frequency ranged from 20 to 30 Hz, with 20 Hz being the most common frequency used. The stimulation intensity was individually adjusted according to each participant’s tolerability (Table 2), and current intensities ranged from 0.1 to 5 mA. In all four RCTs, the control group received sham taVNS with the same duration and frequency of rehabilitation therapy as the active intervention group. In two studies, upper extremity robotic therapy was delivered, and in three studies, conventional rehabilitation therapy was delivered; all studies focused on multiple repetitions of upper extremity movements. Study duration, total number of sessions, and frequency ranged from 10 days to 6 weeks and 10–20 sessions, respectively (Table 2). The Fugl-Meyer upper extremity assessment (FMA-UE) was the primary motor outcome for all studies. Few studies measured additional motor outcomes such as the wolf motor function test (WMFT), action recovery arm test (ARAT), functional independence measure (FIM), Medical Research Council motor power scale, and the modified Tardieu scale (Table 2).

##### Outcomes

**Figure 2:**
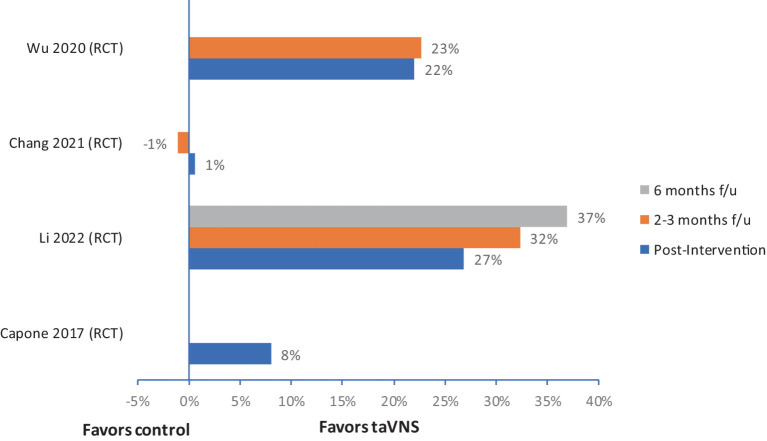
Relative change in FMA-UE with taVNS in people with stoke. f/u, follow-up.

Four out of five studies reported improvement in FMA-U compared to control or baseline. The median (IQR) for mean change in UE-FMA scores from baseline to post-intervention in the VNS group (*n* = 60) was 6.2 (4.8–8.4), and in the control group (*n* = 58) was 3 (2.9–3.5). The relative improvement of FMA scores in three RCTs ranged from 8% to 27% (Figure 2) at post-intervention assessment, and these improvements were boosted at follow–up in two studies (Figure 2) (53, 56, 57). There were no serious adverse events reported in any of the studies. Most of the studies monitored changes in blood pressure and heart rate; no significant changes were noted in these measures.

#### Invasive VNS human stroke studies

Two pilot studies and one pivotal Phase III study using invasive VNS for stroke rehabilitation met our enrollment criteria (27, 28, 33). Only the pivotal trial was powered for efficacy. In total, 146 participants were enrolled. All participants were diagnosed with ischemic stroke between 4 months and 10 years after stroke onset and demonstrated moderate to severe impairment based on FMA-UE or ARAT. In total, 142 (97%) participants completed all treatment sessions. All three studies used Vivistim® Paired VNS System™ as an implant device. The implant surgery was performed under general anesthesia on the left vagus nerve. An overview of the stimulation parameters is given in Table 2. Specific VNS parameters in all three studies were the same using 0.5 s burst VNS (0.8 mA, pulse duration 100 μs, frequency of 30 Hz). While one pilot study used VNS implants only in the active VNS+ rehabilitation group, in the other two studies, VNS was implanted in all participants, including controls. Thus, 134 participants had VNS implantation in these three studies. In-clinic rehabilitation therapy was provided three times per week for 6 weeks (total of 18 sessions) paired with VNS in the active intervention group. Participants in the control group received similar upper limb rehabilitation in all studies, and in two studies, it was combined with sham VNS (Table 2). In the active intervention group, the average number of stimulations during each session ranged between 414 and 444. The two-hour therapy session consisted of stretching and individualized progressive functional tasks targeting movements required to perform activities of daily living such as reach and grasp, etc. Each task was performed for about 10 min, averaging 450 movements per session. After 6 weeks of in-clinic therapy in two studies, participants continued with a 30-min home exercise program over 3 months (27, 33). The primary safety outcome measure was the number of serious adverse events related to the device implantation or rehabilitation therapy with or without VNS.

##### Outcomes

**Figure 3:**
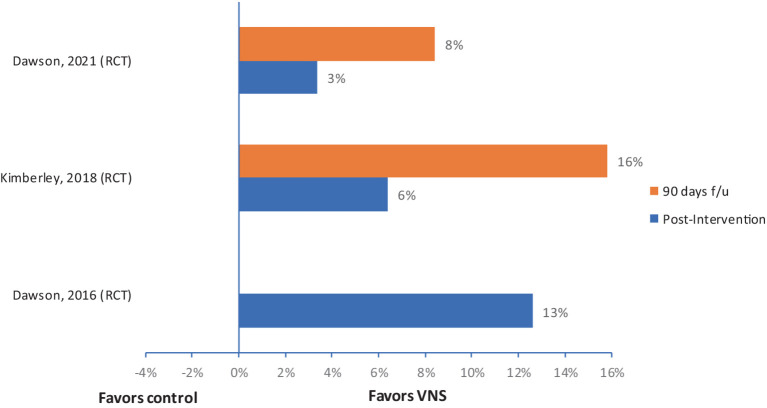
Relative change in FMA-UE with CVNS in people with stoke. f/u, follow-up.

In all three studies, the primary efficacy outcome measure was a change in FMA-UE from baseline to the first day after the completion of in-clinic therapy, and the safety outcome measure was the number of serious adverse events related to the device implantation or rehabilitation therapy with or without VNS. Details on improvement in FMA-UE scores in VNS and control groups are reported in Table 3. Overall median (IQR) improvement in FMA-UE scores in the VNS group (*n* = 70) was 7.6 (6.3–8.2), and in the control group (*n* = 75) was 3 (2.7–4.2). These improvements were not statistically significant in these two studies. However, in these two studies, 67 and 88% of participants achieved a clinically meaningful response on the FMA-UE score in the VNS group compared with controls, in which 36 and 33%, respectively, achieved a clinically meaningful improvement. In the third RCT by Dawson et al., clinically meaningful improvement in FMA-UE was achieved in 23 (47%) participants in the VNS group vs. 13 (24%) participants in the control group (*p* = 0.0098). The relative improvement in FMA-UE in these three studies ranged from 3% to 13% in the active VNS group compared to controls, and this improvement was further accentuated at 90 days follow-up in studies that had a home exercising program (Figure 3).

### Adverse events

The majority of the serious adverse events were post-surgical and related to device implantation. Among 134 participants who had VNS implantation in these three studies, three developed vocal cord palsy (2%), two developed dysphagia (1.4%), one participant had surgical site infection (0.7%), one reported disturbance in taste (0.7%), and 10 participants reported hoarseness of voice (7%) (27, 28, 33). Authors reported all the events were resolved at follow-up. Vocal cord palsy took the longest to resolve, with up to 9 months of recovery in one study (28). There were no serious adverse events with the vagus nerve stimulation protocol. The most commonly reported VNS-related adverse effects were occasional tingling and hoarseness in some participants, which did not persist once the stimulation was aborted.

#### Invasive and non-invasive VNS human SCI studies

There are no published human invasive and non-invasive studies of VNS on motor recovery in SCI. However, we are aware of two centers studying the effects of paired invasive VNS with rehabilitation on motor recovery in people with SCI (NCT05601661, NCT04288245). Results from these studies will provide data on the safety, feasibility, and efficacy of paired VNS on motor recovery in people with SCI.

## Discussion

We aimed to comprehensively review animal and human VNS studies on motor recovery in stroke, TBI, and SCI models/populations. Overall, in 11 studies with rat models of stroke or SCI, upper extremity motor function outcomes consistently improved across both invasive and non-invasive VNS treatment. Notably, all studies demonstrated relatively identical VNS parameters (amplitude, frequency, and duration of stimulus) and intervention duration and frequency. In 10 studies, task training paired with active VNS (up to 9,000 stimulations over 5–6 weeks) improved motor outcomes compared to controls with sham VNS. In these studies, active VNS was delivered only during a successful attempt. Alternative stimulation protocols, including delayed VNS delivered after the rehabilitation therapy and extra VNS delivered at a higher frequency on all trials resulting in >9,000 stims over 5 weeks, appear to have an inconsistent and inferior degree of effect on motor outcomes in comparison to paired VNS during only successful trials. Only one animal SCI study further assessed the importance of triggering VNS on the most successful movements (52). In this study, one group received VNS groups on stronger trials (higher pull force), and another VNS group received VNS only during weaker trials. The group that received VNS during stronger trials had statistically significant improvement in peak pull force post-intervention compared to the control group, but the group that received VNS during weaker trials had no difference in peak pull force compared to controls. Improvement in motor recovery after VNS treatment in animal models was not associated with lesion size change except in one animal study. In the study by Jiang et al., auricular VNS 30 min twice daily for 3 weeks during the acute phase after reperfusion therapy resulted in a reduction in infarct size compared to controls with only reperfusion with or without sham VNS. In this acute VNS stimulation model, additional findings of improved surviving neurons, angiogenesis, and increased expression of neurotrophic and pro-angiogenic factors were present, suggesting a neuroprotection mechanism if applied during the acute phase (47). The improved motor recovery without reducing lesion size in the sub-acute phase was hypothesized secondary to enhancing plasticity in residual motor networks (25, 46, 52).

Four of the five non-invasive human studies demonstrated motor improvements compared to sham control. These improvements were present in three RCTs and compared to the baseline in one pre-and post-intervention study without controls. However, several limitations were noted in these studies, including smaller sample sizes (no power calculations), lack of temporal precision on the application of VNS during rehabilitation training, and heterogeneity of intervention type, frequency, and durations, which limits the interpretation of these study results. The human-invasive VNS studies consistently demonstrated relative motor improvements (Figure 3) but relative improvement varied in each study. Two studies assessing home programs for 90 days were promising for continuing improvements over time with continued in-home exercise programs with VNS. Indeed, in the largest trial of VNS for motor recovery in humans, the findings are promising to enhance motor outcomes compared to traditional therapies. However, the dosing and precision of VNS relative to movement or task, identified as a critical component in animal studies, have not yet been studied in humans.

Due to heterogeneity in study protocols as mentioned above and lack of power in most human studies to find the efficacy, we did not perform a meta-analysis. Instead, we provided relative improvement in outcomes in active VNS group compared to controls for individual RCTs (Figures 2, 3). It is crucial to study the comparison of non-invasive and invasive VNS on motor recovery utilizing a standardized rehabilitation and VNS protocol for a meaningful comparison in future.

We noted no publications assessing VNS in humans with SCI, but we found two active ongoing VNS clinical trials (NCT05601661, NCT04288245) in individuals with chronic (>12 months) cervical spinal cord injury for upper extremity function. VNS studies in individuals with SCI pose additional challenges and require additional considerations. Individuals with cervical SCI often undergo surgical procedures for anterior cervical discectomy and fusion (ACDF) after the SCI, resulting in subsequent scarring in the anterior cervical area, which can complicate electrode implantation on the cervical vagus nerve. Some individuals develop sub-clinical or clinical vocal cord paralysis after ACDF, and VNS implantation surgery is also associated with the risk of vocal cord paralysis. To mitigate this risk, laryngoscopic evaluation of vocal cords is recommended before device implantation. Additionally, autonomic dysfunction in individuals with SCI results in bradycardia, autonomic dysreflexia, and orthostatic hypotension, which warrants the need for safety and feasibility studies prior to rapid translation of this intervention in individuals with SCI.

## Conclusion

Based on our review, we conclude that VNS may enhance the effects of rehabilitation therapy and improve motor outcomes in human stroke populations. Some additional risks are associated with device implantation with the invasive mode of VNS. However, the risk of serious adverse events that lasted for a longer duration is ≤2%. The following studies are needed to address the current gap in the literature: (1) studies to evaluate the temporal precision of VNS with respect to task training, (2) comparative effectiveness studies to examine the effects of non-invasive and invasive VNS compared to control to determine the additional benefit at the risk of vocal cord paralysis, dysphagia, and other risks associated with device implantation in invasive VNS, (3) dosing studies to determine optimal stimulation parameters, frequency, and duration of paired VNS therapy in both clinic and home settings to optimize the outcomes, and (4) studies to assess safety, feasibility, and efficacy of both non-invasive and invasive studies in individuals with TBI and SCI.

## Data availability statement

The original contributions presented in the study are included in the article/Supplementary material, further inquiries can be directed to the corresponding author.

## Author contributions

RK: Writing – review & editing, Writing – original draft, Supervision, Resources, Project administration, Methodology, Investigation, Formal analysis, Data curation, Conceptualization. AM: Writing – review & editing, Writing – original draft, Project administration, Methodology, Funding acquisition, Data curation. AP: Writing – review & editing. NY: Writing – review & editing, Writing – original draft, Supervision, Resources, Methodology, Investigation, Formal analysis, Data curation.
