# Supplementary material for: Neurorehabilitation with vagus nerve stimulation: a systematic review
Source: Front Neurol. 2024 May 30;15:1390217. doi: 10.3389/fneur.2024.1390217 (PMC11169586; doi:10.3389/fneur.2024.1390217)
Supplement: Supplementary file 1 [file Table_1.docx]

| **Study** | **Random sequence generation** | **Deviation from intended intervention** | **Missing outcome data** | **Bias in the measurement of outcome** | **Bias in selection of the reported outcome** | **Overall Bias** |
| --- | --- | --- | --- | --- | --- | --- |
| **Non-Invasive VNS studies** |  |  |  |  |  |  |
| Capone et al. 2017 | Some | Low | Some | Low | Low | some |
| Wu et al. 2020 | Low | Low | Low | Low | Low | Low |
| Chang et al. 2021 | Low | Low | Some | Low | Low | Some |
| Li et al. 2022 | Low | Low | Some | Low | Low | Some |
|  |  |  |  |  |  |  |
| **Invasive VNS studies** |  |  |  |  |  |  |
| Dawson et al. 2016 | Low | Low | Low | Some | Low | Some |
| Kimberley et al. 2018 | Low | Low | Low | Low | Low | Low |
| Dawson et al. 2021 | Low | Low | Low | Low | Low | Low |

**Risk of Bias assessment for randomized control trials (ROBIN-2 tool)**
